# Molecular Mimicry by an F-Box Effector of *Legionella pneumophila* Hijacks a Conserved Polyubiquitination Machinery within Macrophages and Protozoa

**DOI:** 10.1371/journal.ppat.1000704

**Published:** 2009-12-24

**Authors:** Christopher T. Price, Souhaila Al-Khodor, Tasneem Al-Quadan, Marina Santic, Fabien Habyarimana, Awdhesh Kalia, Yousef Abu Kwaik

**Affiliations:** 1 Department of Microbiology and Immunology, College of Medicine, University of Louisville, Kentucky, United States of America; 2 Department of Microbiology, University of Rijeka, Rijeka, Croatia; 3 Department of Biology, University of Louisville, Kentucky, United States of America; Ohio State University, United States of America

## Abstract

The ability of *Legionella pneumophila* to proliferate within various protozoa in the aquatic environment and in macrophages indicates a remarkable evolution and microbial exploitation of evolutionarily conserved eukaryotic processes. Ankyrin B (AnkB) of *L. pneumophila* is a non-canonical F-box-containing protein, and is the only known Dot/Icm-translocated effector of *L. pneumophila* essential for intra-vacuolar proliferation within both macrophages and protozoan hosts. We show that the F-box domain of AnkB and the ^9^L^10^P conserved residues are essential for intracellular bacterial proliferation and for rapid acquisition of polyubiquitinated proteins by the *Legionella*-containing vacuole (LCV) within macrophages, *Dictyostelium discoideum*, and *Acanthamoeba*. Interestingly, translocation of AnkB and recruitment of polyubiquitinated proteins in macrophages and *Acanthamoeba* is rapidly triggered by extracellular bacteria within 5 min of bacterial attachment. Ectopically expressed AnkB within mammalian cells is localized to the periphery of the cell where it co-localizes with host SKP1 and recruits polyubiquitinated proteins, which results in restoration of intracellular growth to the *ankB* mutant similar to the parental strain. While an ectopically expressed AnkB-^9^L^10^P/AA variant is localized to the cell periphery, it does not recruit polyubiquitinated proteins and fails to trans-rescue the *ankB* mutant intracellular growth defect. Direct *in vivo* interaction of AnkB but not the AnkB-^9^L^10^P/AA variant with the host SKP1 is demonstrated. Importantly, RNAi-mediated silencing of expression of SKP1 renders the cells non-permissive for intracellular proliferation of *L. pneumophila*. The role of AnkB in exploitation of the polyubiquitination machinery is essential for intrapulmonary bacterial proliferation in the mouse model of Legionnaires' disease. Therefore, AnkB exhibits a novel molecular and functional mimicry of eukaryotic F-box proteins that exploits conserved polyubiquitination machinery for intracellular proliferation within evolutionarily distant hosts.

## Author Summary

*Legionella pneumophila* is abundantly found in the aquatic environment within various protozoa and can cause a severe pneumonia called Legionnaires' disease when it invades human macrophages in the lung. The ability of *L. pneumophila* to invade and proliferate within macrophages and protozoa is dependent on the translocation of specific proteins into the invaded cell via a specialized secretory device, and these proteins modulate various host cell processes. Of these translocated proteins, AnkB is indispensable for intracellular growth of *L. pneumophila* within macrophages and protozoa. Here we show that AnkB is essential for establishing a favorable intracellular replicative niche by promoting the decoration of the *Legionella* containing vacuole (LCV) with polyubiquitinated proteins. The AnkB effector achieves this by mimicking the action of host cell F-box proteins, a highly conserved component of the SCF ubiquitin ligase complex that is found in both unicellular organisms and mammalian cells. Our study provides new insights into the ability of intracellular pathogens to hijack evolutionarily conserved host cell processes through molecular mimicry to establish a favorable replicative niche within various hosts and to cause disease in mammals.

## Introduction

Intracellular pathogens have evolved with remarkable mechanisms to exploit host cell processes to evade degradation within the lysosomes, which is the first line of defense against microbial invasion. The intracellular bacterial pathogen *Legionella pneumophila* is ubiquitous in natural aquatic environments and man-made water systems, where it replicates within various protozoan hosts [1]. Once transmitted to humans by aerosols, *L. pneumophila* replicates within pulmonary macrophages causing pneumonia [1]. Remarkably, intracellular trafficking and intra-vacuolar proliferation of *L. pneumophila* within amoebae and human macrophages is very similar, at the cellular and molecular levels [1]. Within both evolutionarily distant host cells, *L. pneumophila* evades endocytic fusion and intercepts ER-to-Golgi vesicle traffic to remodel its phagosome into a rough endoplasmic reticulum (RER)-derived vacuole [2],[3]. The Dot/Icm type IV secretion system [4],[5] is required for *L. pneumophila* to modulate various mammalian and protozoan cellular processes through translocation of ∼200 effectors into the host cell, but the role of most of these effectors in the intracellular infection is minimal or not known [2],[3],[6].

The similarity in the intracellular life cycle of *L. pneumophila* within protozoan and mammalian cells suggests that co-evolution of this bacterium with protozoa in the aquatic environment has allowed this bacterium to undergo patho-adaptation and evolution to exploit evolutionarily conserved eukaryotic processes that have enabled this bacterium to proliferate within human macrophages [1]. It is not surprising that bioinformatic genomic analyses of *L. pneumophila* have revealed numerous eukaryotic-like genes, such as ankyrin (*ank*)-encoding genes [7],[8] which have been suggested to be acquired by cross-kingdom horizontal gene transfer. Among ∼200 Dot/Icm-exported effectors, AnkB and SidJ are the only effectors essential for proliferation within the two evolutionarily-distant hosts, mammalian macrophages and protozoa [9]–[11]. In addition to the two Ankyrin domains (ANK), AnkB harbors an F-box domain [9],[10], which is a prevalent domain in eukaryotic proteins known to be involved in polyubiquitination within eukaryotic cells [12],[13].

Ubiquitination of proteins is an ancient and highly conserved eukaryotic post-translational modification process that covalently links a 76-residue ubiquitin polypeptide to a specific protein to target it for proteasomal degradation or to modulate its function in a wide range of important cellular processes [13]. A major group of ubiquitin ligases is the SCF1 (RBX1-CUL1-SKP1) tri-molecular complex that binds the F-box domain [12],[14], which is highly conserved throughout the eukaryotic kingdom, including yeast and the social amoeba *Dictyostelium discoideum* [15],[16]. Substitution of the two conserved LP residues of the F-box domain in unicellular or multi-cellular eukaryotes abolishes polyubiquitination by the SCF1 complex [15],[16]. Polyubiquitination by the SCF1 complex in the social amoeba *D. discoideum*, *Tetryahymena*, *Acanthamoeba*, and yeast appears to function in an identical manner to those of mammalian cells [15]–[17], [18], [19], [20], [21], [22]–[24]. Although members of the F-box family of human proteins typically have a substrate-binding domain such as leucine-rich repeat (LRR) or WD40 [25],[26], the ANK domain has never been described in eukaryotic F-box proteins [12],[13], indicating a novel structure of the microbial F-box AnkB protein.

Many intracellular bacterial pathogens have been shown to exploit or intercept the host polyubiquitination machinery [27]. The *Legionella*-containing vacuole (LCV) is decorated with polyubiquitinated proteins, and the Dot/Icm transport system is essential for this process [28]. We show that AnkB is a non-canonical F-box protein that exhibits molecular and functional mimicry of evolutionarily conserved eukaryotic F-box proteins that enables *L. pneumophila* to hijack a conserved polyubiquitination machinery within mammalian and protozoan cells. Interestingly, AnkB is rapidly translocated into the host cell upon bacterial attachment to the host cell plasma membrane and polyubiquitinated proteins are rapidly recruited to the plasma membrane beneath the sites of bacterial attachment. This molecular and functional mimicry by the F-box domain of AnkB in exploitation of the polyubiquitination machinery within evolutionarily distant host cells is also essential for intrapulmonary bacterial proliferation in the animal model of Legionnaires' disease. The host factor SKP1 interacts with AnkB, and knockdown expression of SKP1 renders the cells non-permissive for intracellular proliferation.

## Results

### The F-box-containing AnkB is essential for acquisition of polyubiquitinated proteins by the LCV within macrophages

**Figure 1:**
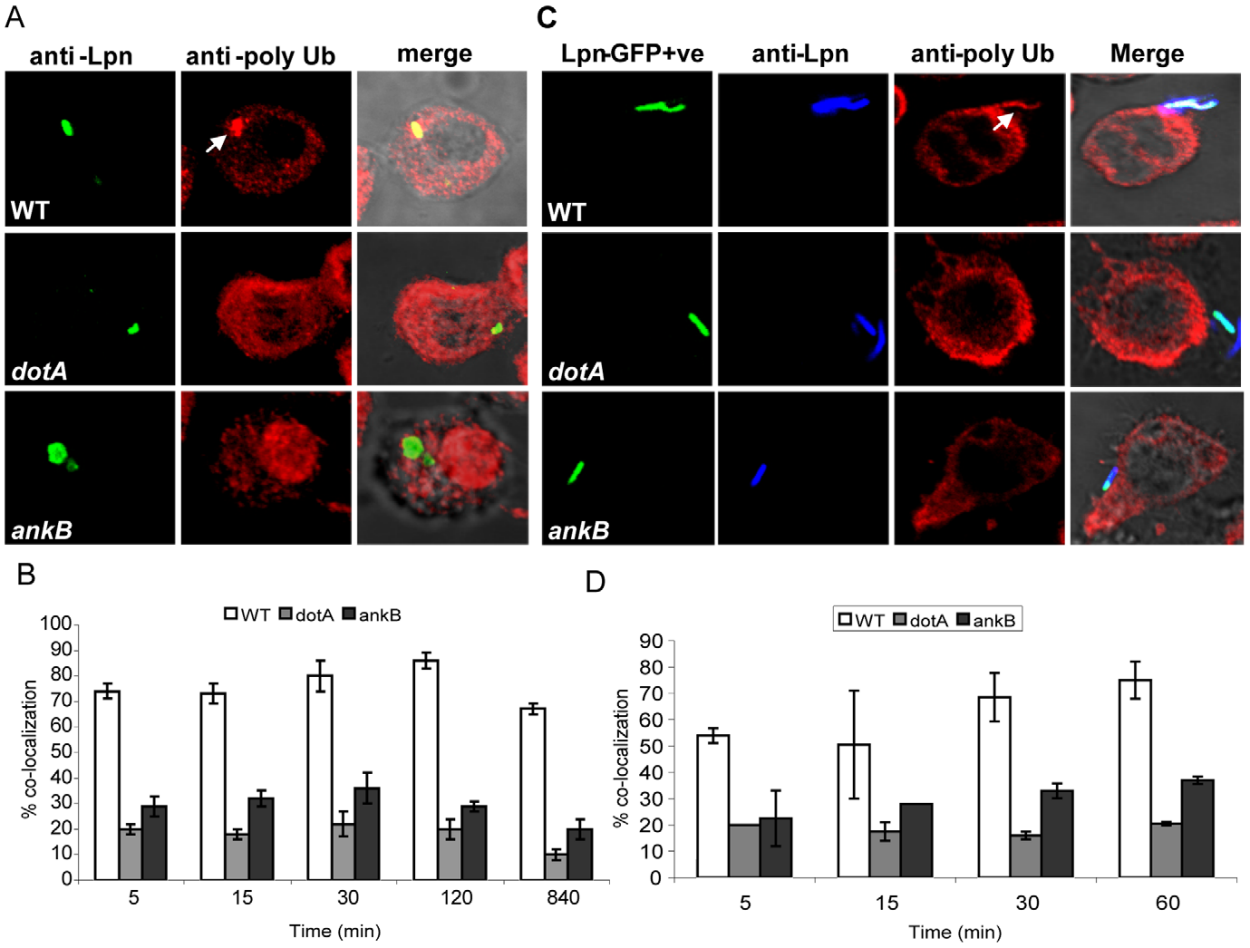
AnkB is essential for recruitment of polyubiquitinated proteins by the LCV and bacterial attachment to the macrophage plasma membrane is sufficient to trigger the recruitment process. U937 cells were infected with the wild type (WT) *L. pneumophila* (Lpn) and the isogenic *dotA* or *ankB* mutants. A) Representative confocal microscopy images of infected U937 cells at 2 h post-infection for co-localization of the LCVs with polyubiquitinated (PolyUb) proteins. The arrow indicates heavy co-localization of polyubiquitin with the WT strain. B) Quantitation of the co-localization during infection at different time points. In panels C and D, the U937 cells were treated with 1 µM cytochalasin D for 30 min prior to infection, to inhibit bacterial entry. The cells were infected with GFP-expressing bacteria for 5, 15, 30 and 60 min. Prior to permeabilization, extracellular *L. pneumophila* were labeled with an anti-Lpn antibody (blue) resulting in a dual labeling of the extracellular bacteria (green and blue). Host polyubiquitin was labeled after permeabilization (red). C) Representative confocal microscopy images that show an extracellular WT *L. pneumophila* bacterium co-localized with high levels of polyubiquitinated proteins (arrow) at the site of bacterial attachment after 5 min of infection. D) Quantitation of the %co-localization of attached bacteria with polyubiquitinated proteins was determined by analysis of 100 attached extracellular bacteria. The data represent analyses of 100 infected cells and are representative of three independent experiments, and the error bars represent standard deviation.

We determined whether the F-box protein AnkB (Fig. S1) mimicked the action of host F-box proteins and recruited polyubiquitinated proteins to the LCV [28]. The wild type strain *L. pneumophila* AA100/130b, the *ankB* mutant, and a *dotA* type IV translocation-defective mutant control were used to infect the U937 human monocytic cell line for 5–120 min and 14 h. At different time intervals post-infection, the cells were labeled using an anti-polyubiquitin antibody and examined by confocal microscopy. The data showed that as early as 5 min post-infection, >70% of the LCVs harboring the WT strain were decorated with polyubiquitinated proteins, while the *ankB* mutant failed to recruit polyubiquitinated proteins, similar to the *dotA* translocation-defective mutant (Fig. 1). Defect of the *ankB* mutant in decorating the LCV with polyubiquitinated proteins was restored by complementation with the WT *ankB* gene on a plasmid (data not shown).

**Figure 2:**
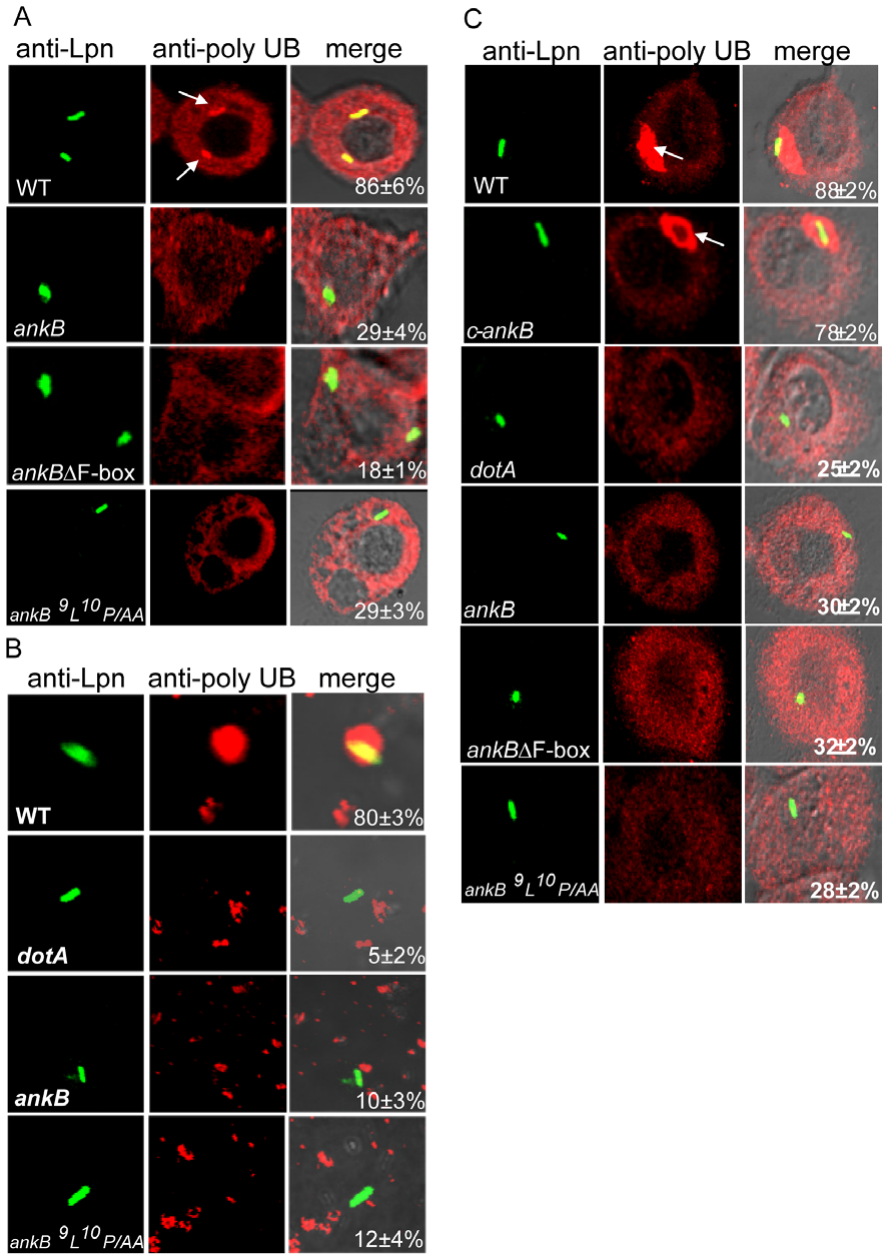
AnkB is essential for recruitment of polyubiquitinated proteins by the LCV in human macrophages. U937 Cells (A and B) or hMDMs (C) were infected with the wild type (WT) *L. pneumophila* (Lpn) and the isogenic *dotA* or *ankB* mutants. The ankB mutant was complemented with WT *ankB* (*c-ankB*) or *ankB* mutant alleles. A and C are representative confocal microscopy images of infected U937 cells and hMDMs, respectively, for co-localization of the LCVs with polyubiquitinated (PolyUb) proteins at 2 h post-infection. The cells were labeled with anti-Lpn antibody (green) and anti-polyubiquitin (red) and then analyzed by confocal microscopy. The arrow indicates heavy co-localization of polyubiquitin with the WT strain. B) Semi-purified LCVs from U937 macrophages after 2 h of infection were labeled as described above for whole cells to determine the frequency of acquisition of polyubiquitinated proteins by the LCV. The numbers in the third columns of all panels are quantitation of the frequency of acquisition of PolyUb proteins by the semi-purified LCV. The data represent analyses of 100 infected cells or LCVs and are representative of three independent experiments.

These observations were further confirmed in semi-purified LCVs in post-nuclear supernatant (PNS) of infected U937 cells. Control LCVs in PNS labeled with anti-*Legionella* antibody prior to permeabilization showed that the majority of the bacteria (95%) were within intact LCVs (data not shown). The data showed that 80% of the purified LCVs harboring the WT strain were decorated with polyubiquitin, while less than 10% of LCVs containing the *ankB* mutant were decorated with polyubiquitin, similar to the *dotA* mutant (Fig. 2). To validate our observations in primary cells, we utilized infection of human monocyte-derived macrophages (hMDMS). Essentially, similar results of polyubiquitination of the LCV were obtained in hMDMs at all time points examined up to 14 h (Fig. 2 and data not shown). Defect of the *ankB* mutant in decorating the LCV with polyubiquitinated proteins was restored by complementation with the WT *ankB* gene on a plasmid (Fig. 2). We conclude that the *L. pneumophila* Dot/Icm-translocated F-box-containing AnkB [9],[10] is essential for acquisition of polyubiquitinated proteins by the LCV within human macrophages.

### AnkB-mediated acquisition of polyubiquitinated proteins by the LCV in *A. polyphaga* and *D. discoideum*

**Figure 3:**
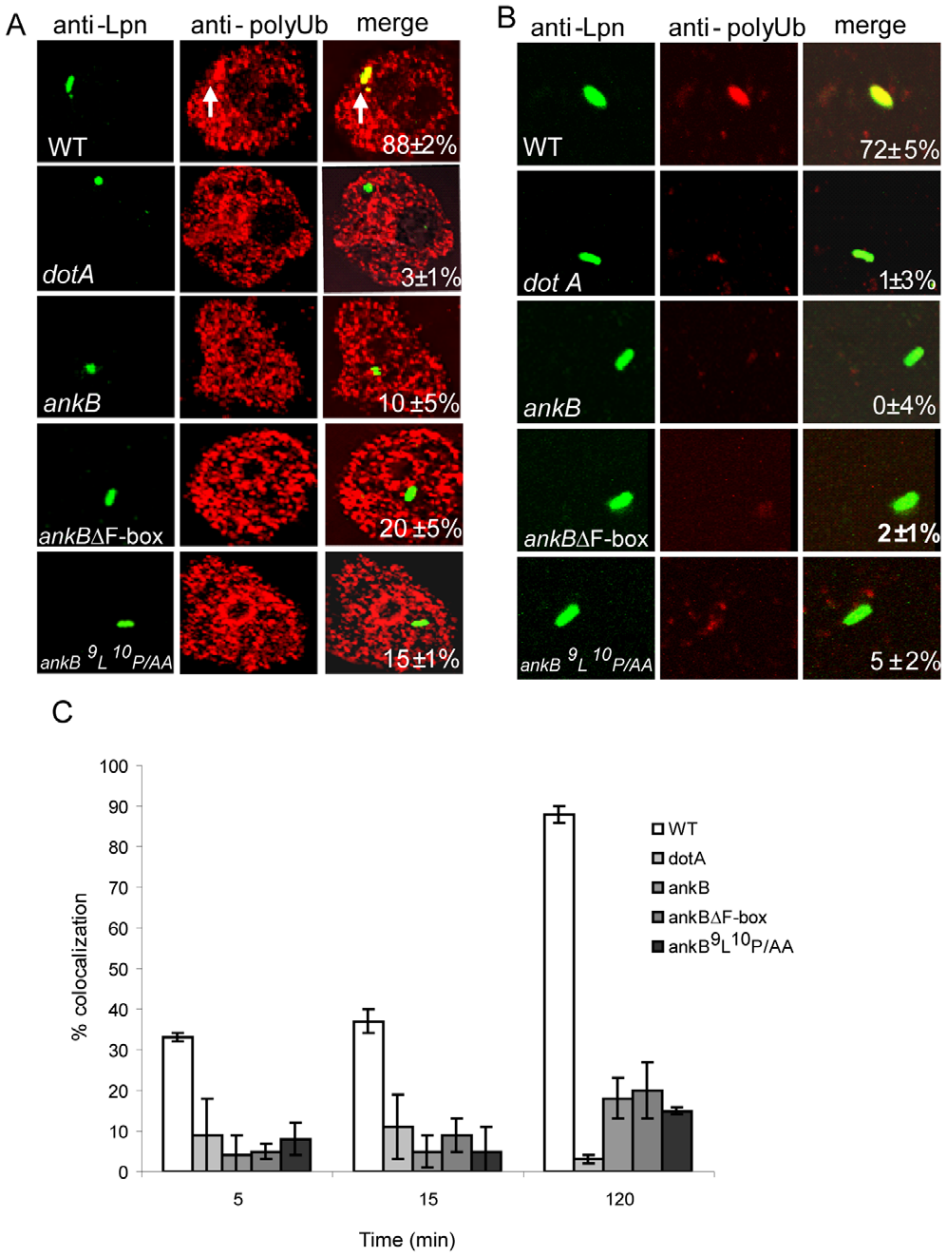
AnkB is essential for acquisition of polyubiquitinated proteins by the LCV in *A. polyphaga*. Representative images of co-localization of the LCVs within *A. polyphaga* cells (A) or in semi-purified LCVs (B) with polyubiquitinated proteins at 2 h post-infection. The cells or LCVs were labeled with anti-Lpn antibody (green) and anti-polyubiquitin (red) and then analyzed by confocal microscopy. The arrow indicates heavy co-localization of polyubiquitin with the WT strain. Quantification of co-localization of the LCVs with polyubiquitinated proteins at 2 h is shown in the merged images in the third column in A and B. C) Quantitation of co-localization of the LCVs with polyubiquitinated proteins at various time points post-infection. The data represent analyses of 100 infected cells or LCVs and are representative of three independent experiments.

Whether the LCV hijacks the polyubiquitination machinery of protozoan hosts is not known. Therefore, we determined whether the LCV within *Acanthamoeba polyphaga* and the social amoeba *Dictyostelium discoideum* was decorated with polyubiquitinated proteins and whether the Dot/Icm system type IV secretion system and the AnkB effector were involved in this process. The data showed that similar to human macrophages, the LCVs harboring the wild type strain with *A. polyphaga* (Fig. 3) and *D. discoideum* (Fig. S2) acquired polyubiquitinated proteins as early as 5 min post-infection, while the *ankB* mutant failed to recruit polyubiquitinated proteins, similar to the *dotA* mutant.

These observations were further confirmed in semi-purified LCVs in post-nuclear supernatant (PNS) of infected *A. polyphaga* cells at 2 h post-infection. Control LCVs in PNS labeled with anti-*Legionella* antibody prior to permeabilization showed that the majority of the bacteria (95%) were within intact LCVs (data not shown). The data showed that the wild type strain purified LCVs were heavily decorated with polyubiquitinated proteins, while LCVs of the *dotA* and *ankB* mutants were both severely defective in acquisition of polyubiquitinated proteins (Fig. 3). We conclude that the AnkB effector is essential for acquisition of polyubiquitinated proteins by the LCV within protozoan cells, similar to human macrophages.

### Translocation of AnkB and recruitment of polyubiquitinated proteins upon attachment of *L. pneumophila* to macrophages and *A. polyphaga*

Due to the rapid recruitment of polyubiquitinated proteins to the LCV within macrophages and amoebae, we hypothesized that the bacteria might initiate the process upon attachment to the host cell membrane. To test this hypothesis, macrophages were pre-treated with cytochalasin D to block bacterial entry, and the extracellular bacteria were labeled with an antibody prior to permeabilization to differentiate them from intracellular bacteria. Interestingly, within 5 minutes of bacterial attachment, >50% of the WT attached bacteria recruited polyubiquitinated proteins to their attachment sites at the macrophage plasma membrane but the *ankB* mutant was defective, similar to the *dotA* mutant (Fig. 1). Similar results were also obtained in *A. polyphaga* (Fig. S3). We conclude that upon attachment of *L. pneumophila* to human macrophages and *A. polyphaga*, AnkB rapidly recruits polyubiquitinated proteins to the host plasma membrane beneath the sites of bacterial attachment.

**Figure 4:**
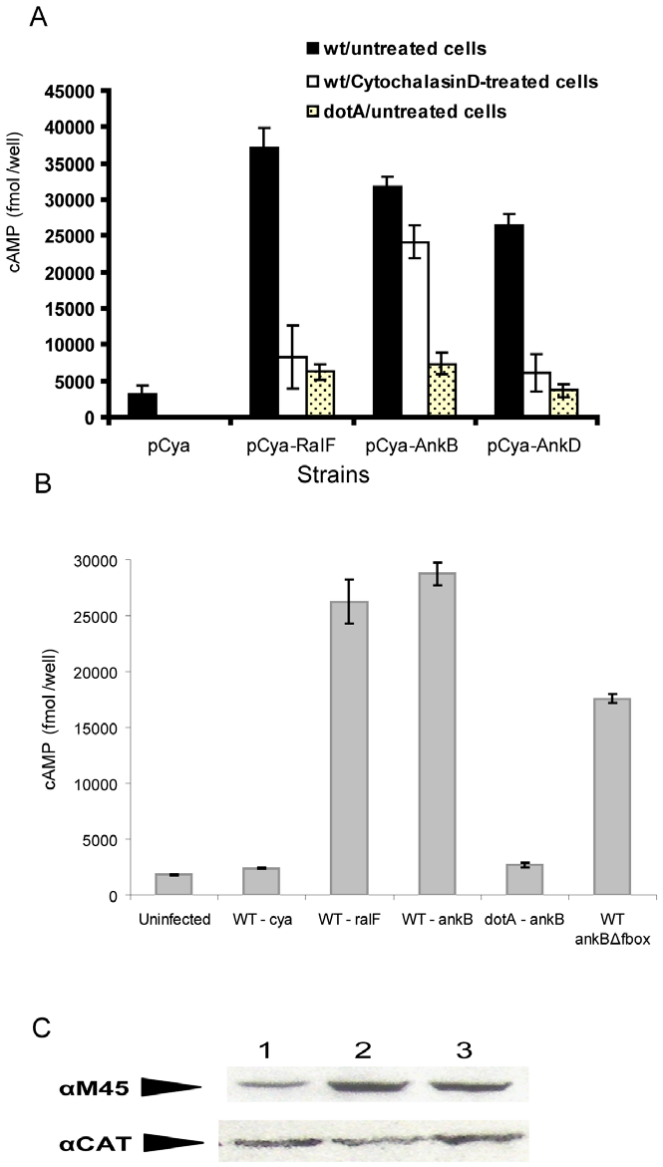
Translocation of AnkB by attached extracellular bacteria and efficient translocation of the AnkBΔF-box reporter by intracellular bacteria. A) Translocation of AnkB into U937 cells by attached WT or dotA mutant extracellular bacteria was determined at 30 min post-infection. Strains harbored either empty vector (pCya) or adenylate cyclase RalF, AnkB or AnkD fusions (pCya-Ralf, pCya-AnkB and pCya-AnkD). B) Translocation of the reporter constructs by intracellular bacteria at 2 h post-infection. C) Equivalent expression of the AnkB-Cya fusion alleles in *L. pneumophila* was determined by immunoblots of Cya-AnkB fusions expressed in *L. pneumophila*. Protein derived from equivalent numbers of bacteria (1×10^8^) were loaded onto a SDS-PAGE gel, and Cya fusion proteins were detected by Western blots probed by an α-M45 antibody, recognizing the N-terminal M45 epitope on all Cya fusions. The blots were re-probed with anti-CAT antibodies, which showed equivalent expression of another protein encoded on the same reporter plasmid. Lanes 1: WT *cya-ankB*; 2: *dotA cya-ankB*; 3: WT *cya*-*ankB*Δ*F-box*. Data points are the average cAMP concentration per well for a representative experiment performed three times in triplicate. Error bars represent standard deviation.

Our data above led us to hypothesize that AnkB was rapidly translocated into the host cell by attached extracellular bacteria. To test this hypothesis, we examined whether AnkB was translocated into macrophages prior to bacterial internalization. We utilized the calmodulin-dependent adenylate cyclase-AnkB (CyaA-AnkB) reporter system [10], which becomes active only after translocation into the host cell, to quantify translocation of the AnkB protein to the host cell. For controls of Dot/Icm-translocated effectors that are only translocated by intracellular and not extracellular bacteria, we used RalF-CyaA and AnkD-CyaA fusions [7]. The results showed that in contrast to the RalF effector and AnkD controls, AnkB was efficiently translocated by attached extracellular bacteria (Fig. 4A). These results are consistent with our data above that showed extracellular bacteria recruited polyubiquitinated proteins to the cytosolic face of the plasma membrane beneath the sites of bacterial attachment.

### Role of the F-box domain of AnkB in acquisition of polyubiquitinated proteins within human macrophages and *A. polyphaga*

The F-box domain is highly conserved through evolution and the two characterized *D. discoideum* F-box proteins harbor a highly conserved F-box [19],[22]. In addition, the LP conserved residues of the F-box domain, which are essential for binding of eukaryotic F-box proteins to the cellular factor SKP1, are also conserved from *D. discoideum* to mammals [15],[16]. To analyze the role of the F-box domain of AnkB in decorating the LCV with polyubiquitinated proteins, we engineered a mutant allele of *ankB* with an in-frame deletion of the F-box domain (*ankBΔF-box*) (Fig. S1). In addition, the conserved ^9^L^10^P residues of the F-box domain were substituted by alanine residues (*ankB-^9^L^10^P/AA*) (Fig. S1). Macrophages and *A. polyphaga* were infected as described above by the wild type strain, the *ankB* mutant, or the *ankB* mutant harboring plasmids containing the WT *ankB* or the *ankBΔF-box* or the *ankB-^9^L^10^P/AA* allele. The data showed that within U937 human macrophages, hMDMs, and *A. polyphaga* the LCVs harboring the *ankB* mutant complemented with the *ankBΔF-box* or the *ankB-^9^L^10^P/AA* allele failed to become decorated with polyubiquitinated proteins, similar to the *ankB* null mutant or the *dotA* mutant control (Fig. 2 and 3). The LCVs harboring the *ankB* mutant complemented with the WT *ankB* allele were decorated with polyubiquitinated proteins, similar to the WT strain (Fig. 2 and 3). These results were further confirmed in semi-purified LCVs harboring the various strains within macrophages or *A. polyphaga* (Fig. 2 and 3). These data show that the F-box domain of AnkB and its two conserved LP residues are essential for acquisition of polyubiquitinated protein by the LCV within the two evolutionarily distant host cells. These data indicate that AnkB may mimic the function of conserved cellular F-box proteins to hijack the ubiquitination machinery of evolutionarily distant hosts.

### Role of the F-box domain of AnkB in intracellular proliferation within human macrophages

To determine the role of the F-box domain in intracellular proliferation in macrophages, the *ankB* mutant was complemented by the *ankBΔF-box* or the *ankB-^9^L^10^P/AA* construct on a plasmid. The data showed that complementation of the *ankB* mutant with the respective wild type gene on a plasmid fully restored intracellular growth within hMDMs to levels similar to the wild type strain (Fig. S4). In contrast, complementation of the *ankB* mutant with the *ankBΔF-box*, or the *ankB-^9^L^10^P/AA* allele failed to restore the intracellular growth defect of the *ankB* mutant in hMDMs. Therefore, the F-box domain of AnkB and its two LP conserved residues are essential for decoration of the LCV with polyubiquitinated proteins and for intracellular proliferation within macrophages.

Translocation of AnkB into the host cell is essential for its crucial role in intracellular proliferation [9],[10]. Therefore, it was essential to confirm that the failure of the *ankBΔF-box* allele to restore to the *ankB* null mutant intracellular proliferation and recruitment of polyubiquitinated proteins was not due to a defect in translocation. We utilized the Calmodulin-dependent adenylate fusion assay to determine translocation of the truncated AnkBΔFbox variant. Immunoblot analyses of bacterial cell lysates showed equivalent expression of the Cya-AnkB fusions among all the tested strains (Fig. 4). The data showed that the AnkBΔFbox was efficiently translocated into the host cell, albeit at slightly reduced levels compared to the WT AnkB (Fig. 4). Thus, the defect in polyubiquitination and intracellular proliferation of the AnkBΔFbox variant was not likely to be due to reduced expression or stability of the AnkBΔFbox variant protein.

### Ectopic expression of AnkB and its distribution in mammalian cells

**Figure 5:**
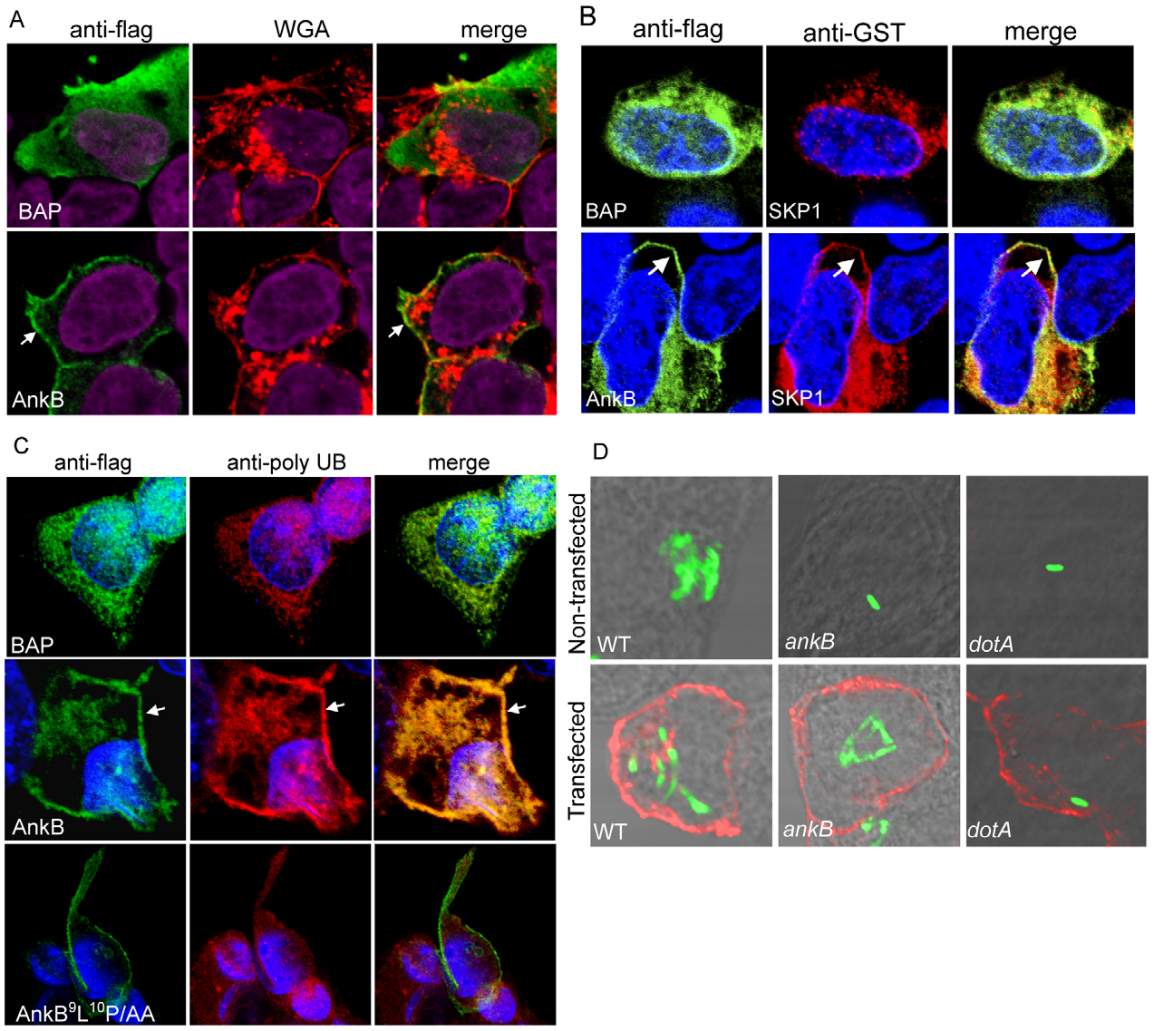
Ectopically expressed 3X-Flag AnkB is distributed at the periphery of HEK-293 cells, co-localizes with the three SCF components and poly-ubiquitinated proteins, and restores intracellular growth to the *ankB* mutant. A) Representative confocal images of HEK-293 cells transfected with plasmids encoding 3X-Flag BAP or 3X-flag AnkB. At 24 h following transfection, cells were labeled using a mouse anti-Flag (green) and WGA conjugated to Alexa Fluor-647 (red). Nuclei were stained with DAPI (purple). The white arrow indicates peripheral localization of 3X-Flag AnkB. B) Representative confocal images of HEK-293 cells that were co-transfected with plasmids encoding 3X-flag AnkB or BAP and SKP1-GST. Cells were stained with anti-Flag (green) and anti-GST (red) and nuclei were visualized with DAPI (blue). Arrows indicate peripheral co-localization of AnkB and SKP1. C) 3X-Flag AnkB co-localizes with polyubiquitinated proteins at the cell periphery. At 24 h following transfection cells were labeled using a rabbit anti-flag antibody (green) and a mouse anti-polyubiquitin antibody (red). Nuclei were labeled with DAPI (blue). The white arrow indicates heavy co-localization of 3X-Flag AnkB with polyubiquitin. D) Representative confocal images at 12 h post-infection of HEK-293 cells transfected for 24 h with a plasmid encoding 3X-Flag AnkB. The cells were labeled with an anti-Lpn antibody (green) and an anti-Flag antibody (red). The data are representative of three independent experiments.

Although AnkB is clearly translocated into the host cell by the Dot/Icm system, we have been unable to detect AnkB during infections using AnkB-specific antibodies (data not shown), indicating that the protein is either extremely unstable or translocated by the bacterium at undetectable concentrations. Several other Dot/Icm effectors such as LidA and LubX [29],[30] have also been shown to be undetectable, which may ensure limited interference with host cell processes without compromising viability of the host cell. To overcome this limitation and to elucidate the distribution of AnkB in the host cell, we ectopically expressed a 3X-flag AnkB fusion construct in the HEK-293 human cell line. Ectopic expression was not toxic to the cells, which was further confirmed by our ability to generate stable HEK293 cell transfectants (Fig. 5A, data not shown). Transient transfection of HEK-293 cells with a plasmid encoding 3X-Flag AnkB revealed a striking localization of this protein to the periphery of the cell. In contrast, the control protein 3X-Flag-bacterial alkaline phosphatase (BAP) was distributed throughout the cytosol (Fig. 5A). Localization of the 3X-Flag AnkB to the periphery of the cell, beneath or at the inner layer of the plasma membrane, was further supported by labeling the plasma membrane with wheat germ agglutinin (WGA) prior to permeabilization and fixation, which revealed close association of AnkB to the periphery of the cell, or the inner layer of the plasma membrane (Fig. 5A). In addition, AnkB was also observed as a few punctate spots within the cytosol (Fig. 5). Substitution of the conserved ^9^L^10^P residues of the F-box domain to AA did not impair localization of AnkB to the cell periphery (Fig. 5C). Similar results were obtained using AnkB-GFP fusions (data not shown).

To determine whether AnkB recruits the host SKP1 protein to its peripheral localization sites in the cells, we co-transfected HEK293 cells with plasmids encoding 3X-flag AnkB and SKP1-GST (Fig. 5B). There was a clear and distinct co-localization of SKP1 at the cell periphery where AnkB was concentrated and preferentially localized (Fig. 5B).

Since AnkB is essential for recruitment of polyubiquitinated substrates to the LCV, we determined whether ectopic expression of 3X-Flag AnkB and its recruitment of host SKP1 to the periphery of the cell also altered distribution of polyubiquitinated proteins in the host cell. Strikingly, strong co-localization of 3X-flag AnkB and polyubiquitinated proteins was observed at the periphery of transfected HEK-293 cells (Fig. 5C). In contrast, 3X-flag AnkB-^9^L^10^P/AA failed to co-localize strongly with polyubiquitinated proteins, similar to the BAP control (Fig. 5E), despite its similar distribution in the cell to that of the native AnkB protein. These data indicate that the two LP conserved residues of the F-box domain do not affect localization of the AnkB protein in HEK-293 cells, but are crucial for the ability of AnkB to hijack the host ubiquitination machinery. Thus, ectopically expressed AnkB recruits host SKP1 protein and acts as a platform for acquisition of polyubiquitinated proteins at the periphery of the cell.

We utilized laser scanning confocal microscopy to determine trafficking and potential co-localization of ectopically expressed AnkB with endosomal, lysosomal, Golgi and ER compartments using the specific markers Lamp2, cathepsin D, GM130 or P58k and KDEL, respectively. The data showed that there were no significant differences in association of the above markers with AnkB compared to the negative control (student *t*-test, *p*>0.5) (data not shown). Localization of ectopically expressed AnkB to the periphery of the cell, or at the inner layer of the plasma membrane and its lack of localization to organelles and vesicles tested is consistent with the function of AnkB in events independent of trafficking of the LCV and its evasion of endocytic fusion [10].

### Ectopic expression of AnkB in mammalian cells rescues growth of the *ankB* mutant

Since the *ankB* mutant is severely defective in intracellular proliferation, we determined whether ectopically expressed 3X-Flag AnkB and its recruitment of polyubiquitinated proteins to the periphery of the cell would be sufficient to restore intracellular growth to the *ankB* mutant. The 3X-flag AnkB-transfected HEK-293 cells were infected for 1 h and the number of intracellular bacteria was determined at 12 h using single cell analysis by microscopy. At 2 h post-infection most cells harbored a single organism (data not shown). The data revealed that indeed ectopically expressed 3X-Flag AnkB restored intracellular growth to the *ankB* mutant at 12 h post-infection, similar to wild type bacteria in transfected or non-transfected cells (Fig. 5D, Fig. S5). Importantly, the *ankB* mutant failed to replicate in un-transfected cells or cells transfected with the 3X-Flag BAP control (Fig. 5D, Fig. S5). Despite the trans-rescue of the *ankB* mutant for its defect in intracellular proliferation in cells expressing AnkB at high levels, the AnkB protein was not detected on the LCV. These data indicate that AnkB may function at a distance from the LCV to hijack the polyubiquitination machinery to enable intracellular proliferation. However, it can not be excluded that possible rapid turnover of AnkB on the LCV may preclude its detection.

### Host SKP1 interacts with AnkB *in vivo* and is essential for intracellular proliferation of *L. pneumophila*

**Figure 6:**
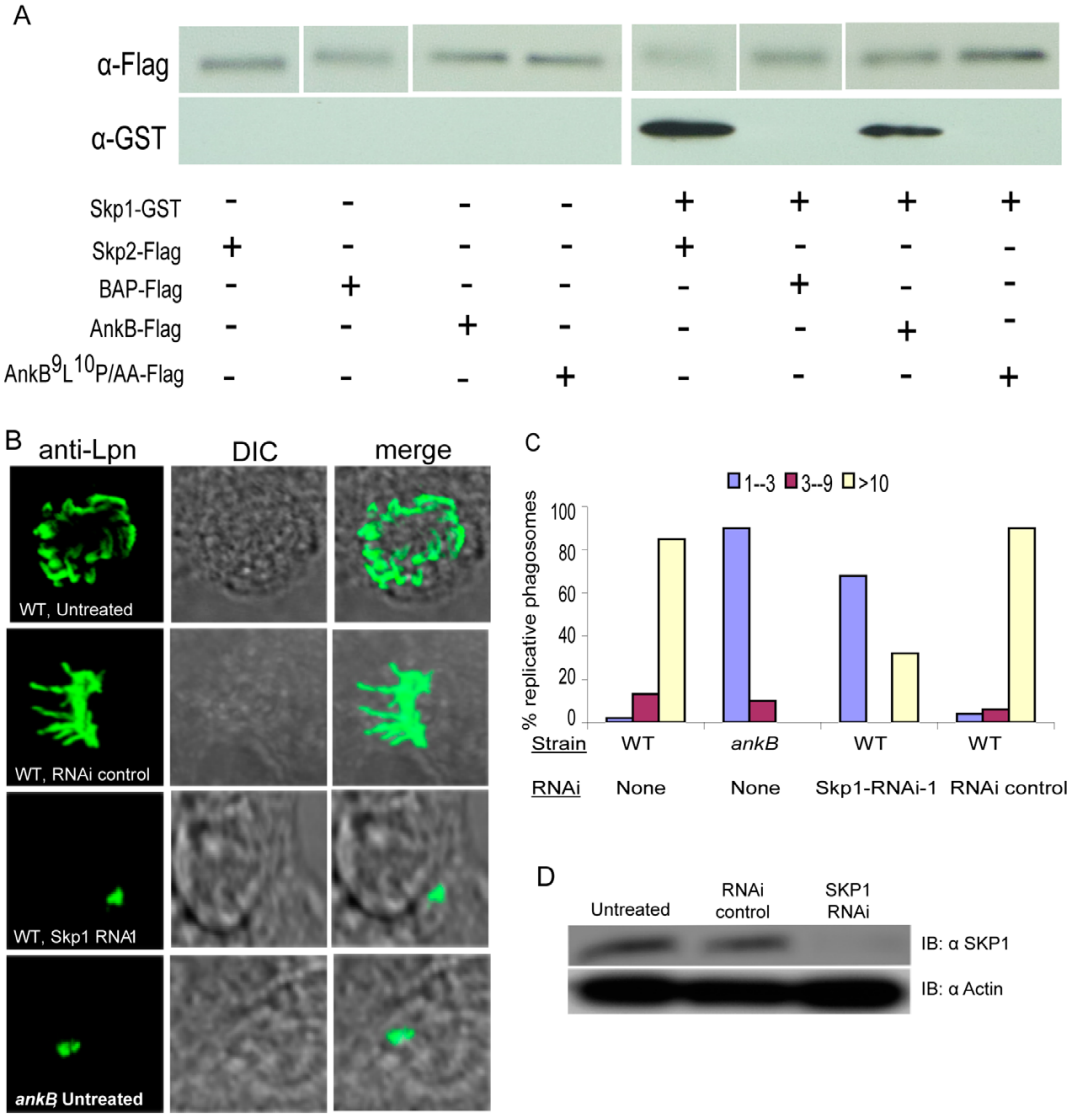
The F-box AnkB protein mimics host F-box proteins by interacting with host SKP1 in vivo and SKP1 is essential for intracellular bacterial proliferation. A) In vivo co-immunoprecipitation of AnkB and SKP1 in co-transfected cells. HEK-293 cells were co-transfected with plasmids encoding 3X-Flag AnkB, AnkB^9^L^10^P/AA, BAP or SKP2 and SKP1-GST. Total protein input in total cell lysate was equivalent in all the lanes (not shown). Cell lysates were purified using anti-flag resin and the resulting supernatants were analyzed by immunoblotting with antibodies against Flag and GST. B) Representative confocal images of HEK-293 cells that were untreated or transfected with RNAi followed by infection and labeling of the bacteria at 16 h to score replicative vacuoles. C) Quantitative analyses of the formation of replicative vacuoles were based on examination of 100 infected cells. The different color bars represent the number of bacteria/vacuole. The data represent analyses of the mean and standard deviations of 100 infected cells, and are representative of two independent experiments. D) Knockdown expression of SKP1 protein by siRNA silencing in HEK-293 cells was confirmed by Western blot of HEK-293 cell lysates probed with an anti-SKP1 antibody. The blots were re-probed with anti-actin antibodies, which showed equivalent quantities of proteins were loaded onto the gel. The results are representative of three independent experiments.

To determine whether AnkB mimicked a host F-box protein and interacted directly with the host factor SKP1, we performed in vivo co-immunoprecipitation. We co-transfected HEK-293 cells with plasmids encoding 3X-Flag tagged AnkB, AnkB^9^L^10^P/AA, BAP or SKP2 and SKP1-GST. The transfected cells were lysed and reciprocal co-immunoprecipitation with anti-flag agarose and anti-GST agarose was performed (Fig. 6A, data not shown). As expected, our data showed that SKP1 and SKP2 co-precipitated whilst SKP1 and BAP did not co-precipitate. Importantly, our data showed that AnkB and SKP1 specifically co-precipitated (Fig. 6A). To test the specificity of this interaction and the direct role of the F-box domain in binding to SKP1, we analyzed if 3X-Flag AnkB^9^L^10^P/AA would co-precipitate with SKP1-GST. The data were very clear that the 3X-Flag AnkB^9^L^10^P/AA did not interact with SKP1-GST (Fig. 6A). This was consistent with data above using scanning confocal microscopy of cells co-transfected with plasmids encoding 3X-Flag AnkB and SKP-GST, where distinct co-localization of SKP1 with AnkB at the cell periphery was detected (Fig. 5B). No specific co-localization with SKP1 and BAP control was observed around the cell periphery (Fig. 5B). Taken together, these results show that AnkB specifically interacts with the host SKP1 *in vivo,* and this interaction absolutely requires the functional F-box domain of AnkB.

To determine whether the host SKP1 function was required for intracellular proliferation of *L. pneumophila*, 3 different SKP1-specific RNAi were transfected separately into HEK293 cells. There was knock-down in the expression of SKP1 by RNAi-1, -2 or a combination of both but viability of the cells was not affected by any of the RNAi used (Fig. 6D and data not shown). However, RNAi-3 or control RNAi had no detectable effect on SKP1 expression by 48 h (Fig. 6D). Two days after transfection, cells were infected with the WT strain *L. pneumophila* or the *ankB* mutant. Single cell analysis by microscopy was performed at 12 hours post-infection to determine the effect on bacterial proliferation at the single cell level (Fig. 6B, C). Silencing of SKP1 by RNAi-1, RNAi-2, or a combination of both completely blocked intracellular replication of *L. pneumophila*, which was reminiscent of the defect of the *ankB* null mutant in non-transfected cells (Fig. 6B, C and data not shown). In contrast, in the non-transfected cells, or cells transfected with control RNAi or RNAi-3, there was no effect on intracellular replication (Fig. 6B, C and data not shown). We conclude that host SKP1 is essential for intracellular replication of *L. pneumophila*. This supports the evidence that the *L. pneumophila* AnkB F-box protein mimics the function of eukaryotic F-box proteins to hijack the host polyubiquitination machinery, which is essential for intracellular proliferation of *L. pneumophila* within mammalian and protozoan cells.

### AnkB is essential for intrapulmonary proliferation in the mouse model of Legionnaires' disease

The role of Dot/Icm-translocated effectors of *L. pneumophila* in intrapulmonary proliferation in animal models has never been examined for any effector. When groups of 10 A/J mice were infected with 10^9^ CFUs of the WT strain or the *ankB* mutant to determine lethality by high dose infection, 100% of the WT strain-infected mice succumbed to infection within 24–48 h after inoculation, while 100% survival for the *ankB* mutant-infected mice was observed up to 7 days. When mice were infected by 10^8^ CFUs, 70% mortality was exhibited by 24–72 h after infection with the WT strain, but 100% survival was observed for the *ankB* mutant-infected mice.

**Figure 7:**
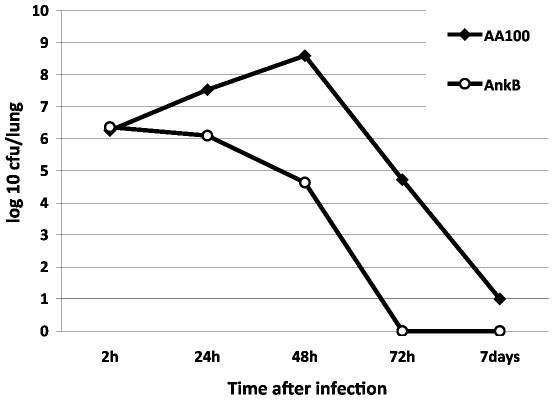
The *L. pneumophila ankB* mutant is defective in the A/J mouse model of Legionnaires' disease. Three A/J mice for each time point were infected with 10^6^ CFUs of *L. pneumophila* WT strain or the *ankB* mutant. After 2 h, 1 day, 2, 3 and 7 days of infection, three mice were sacrificed and lungs were obtained, homogenized and dilutions were plated on agar plated for CFU enumeration after incubation for 72–96 h. The results are the mean of 3 mice/time point. These results are representative of two independent experiments.

To determine whether AnkB was required for intrapulmonary proliferation of *L. pneumophila*, we infected A/J mice with 10^6^ CFUs of *L. pneumophila*. Proliferation of *L. pneumophila* in the lungs of infected mice was assessed by enumeration of the CFUs in lung homogenates after 24 h, 48 h, 72 h and 7 days post-infection. While there was robust intrapulmonary proliferation by the WT strain by 24–48 h, the data were very clear that there was no detectable intrapulmonary proliferation for the *ankB* mutant (student *t*-test, *p*<0.001) (Fig. 7). By 48 h post-infection, at least 2000-fold less bacteria were recovered from lungs infected with the *ankB* mutant compared to the WT strain (Fig. 7). Due to our repeated observations of the rapid loss of plasmids by the wild type strain AA100 or its derivative mutants in mouse lungs, complementation in mouse lungs was not achieved. We conclude that the function of AnkB in recruitment of polyubiquitinated proteins to the LCV is essential *in vivo* for intrapulmonary proliferation of *L. pneumophila* in the mouse model of Legionnaires' disease.

## Discussion

*L. pneumophila* is an environmental bacterium that is believed to have evolved through interaction with various protozoa to acquire mechanisms to allow them to proliferate within mammalian pulmonary macrophages, causing pneumonia. This microbial adaptation to the intracellular life within protozoa and mammalian cells indicates microbial manipulation of eukaryotic cellular processes that are conserved through evolution. Among ∼200 Dot/Icm-exported effectors of *L. pneumophila*, AnkB is the only effector that plays a major role in intracellular proliferation within both protozoan and mammalian cells [10]. This indicates that the host target of AnkB is an evolutionarily conserved process in mammalian and protozoan cells. Comparison of the amino acid sequences for the two characterized *D. discoideum* F-box proteins FbxA and MEKKα to mammalian F-box proteins reveals identical sequences in the conserved F-box motif, including the conserved LP residues (data not shown). The F-box domain of the *L. pneumophila* AnkB protein also harbors an identical sequence to the F-box motif and has the conserved LP residues. We show that the F-box domain of AnkB exhibits molecular and functional mimicry of eukaryotic F-box proteins in exploitation of a conserved polyubiquitination machinery [31] to allow microbial parasitism of evolutionarily distant hosts. Our data show that the F-box of AnkB and its two highly conserved L^9^P^10^ residues are essential for hijacking the polyubiquitination machinery for intracellular proliferation. This indicates that AnkB exhibits molecular and functional mimicry of an evolutionarily conserved eukaryotic F-box protein and that AnkB has conserved eukaryotic targets in mammalian and protozoan cells, which allows *L. pneumophila* to hijack the ubiquitination machinery of the evolutionarily distant host cells. The *L. pneumophila* published genomes indicate that there are at least two other F-box proteins encoding genes but the roles of these in infection are not known [32]–[35]. In addition, a U-box-containing E3 ligase of *L. pneumophila* has been reported but does not play a role in polyubiquitination of the LCV [30]. However, the profound effect of AnkB on polyubiquitination of the LCV clearly indicates a major role for AnkB in this process. We have recently identified tremendous heterogeneity in acquisition of polyubiquitinated proteins by the LCV harboring various clinical and environmental isolates of *L. pneumophila* (Price et al, unpublished data), which indicates potential redundancy for the function of AnkB in various isolates, such as the Lp02 strain [10],[32]. Functional redundancy among Dot/Icm effectors is very common and is thought to allow parasitism of various unicellular hosts in the aquatic environment [2].

Interestingly, the F-box family of human proteins typically have a leucine-rich repeat (LRR) or WD40 protein-protein interaction domains, which bind substrates and link them to the SCF1 ubiquitin ligase tri-molecular complex that is bound to the F-box domain [25],[26]. However, the ANK domain has never been found in eukaryotic F-box proteins [12],[13]. This would render the bacterial F-box-containing AnkB to have a non-canonical or novel structure among the large family of F-box proteins [12],[13]. Interestingly, co-expression of AnkB and SKP1 in cells results in co-localization of the two proteins at the periphery of the cell where polyubiquitinated proteins are recruited, and this is sufficient to trans-rescue the defect of intracellular proliferation of the *ankB* mutant. Importantly, the host SKP1 interacts directly and specifically with AnkB *in vivo*, and the specificity of this interaction has been demonstrated by failure of the AnkB-^9^L^10^P/AA variant to interact with SKP1. Importantly, this interaction is essential for bacterial proliferation, since knockdown expression of SKP1 renders the cells non-permissive for intracellular proliferation of the wild type strain. We conclude that the AnkB-SKP1 interaction and its role in exploitation of the host polyubiquitination machinery is essential for intracellular bacterial proliferation.

Recruitment of polyubiquitinated proteins to the phagosome of intracellular pathogens has been described only for *L. pneumophila*. The quantity, the kind of linkages involved in polyubiquitination, or the identity of the proteins that are polyubiquitinated on the LCV remain to be elucidated. In addition, other components of the SCF1 complex that interact with AnkB need to be identified. Future proteomic studies on these aspects of the LCV to identify these processes would be intriguing. Taken together, we propose a model in which the F-box domain of AnkB interacts with the host SKP1, while protein substrates to be polyubiquitinated are recruited by the two ANK domains of AnkB. The identity of these substrates is currently under investigation.

In summary, we have characterized an essential role for a structurally novel non-canonical F-box translocated effector in a mammalian intracellular bacterial pathogen. This *L. pneumophila* non-canonical F-box effector exhibits molecular and functional mimicry of an evolutionarily-conserved F-box protein in recruitment of the host polyubiquitination machinery to the bacterial vacuole, which is essential for intra-vacuolar proliferation within mammalian and protozoan cells and is essential for manifestation of pulmonary disease in the animal model. The host SKP1 factor interacts directly and specifically with AnkB, and knockdown of its expression renders the cells non-permissive for intracellular proliferation. Our data provide the first description of a bacterial non-canonical F-box protein and its molecular and functional mimicry of evolutionarily conserved F-box proteins to exploit an evolutionarily conserved polyubiquitination machinery to enable intra-vacuolar proliferation within evolutionarily distant hosts and to cause pulmonary disease in mammals.

## Materials and Methods

### Ethics statement

All animal work in this manuscript has been performed after approval by the institutional IACUC committee who follows the US federal guidelines for the use and humane handling of animals in research.

### Bacterial strains, cell cultures and infections

*L. pneumophila* serogroup I parental strain AA100/130b and the mutants *dotA, ankB,* and complemented *ankB* mutants were grown as described previously [10]. *Escherichia coli* strain DH5α was used for cloning purposes. Isolation and preparation of human monocyte-derived macrophages (hMDMs) and maintenance of the macrophage-like U937 cells were performed as previously described [9]. Cultures of *A. polyphaga* and *D. discoideum* were performed as described previously [9],[10].

For intracellular proliferation studies, infections were performed as we described previously [9]. Briefly, macrophages were infected at a multiplicity of infection (MOI) of 10 for 1 h followed by treatment with 50 µg/ml gentamicin for 1 h to kill extracellular bacteria. At each time point, the macrophages were lysed and dilutions were plated on agar plates. For single cell analysis studies, infections were performed as we described above and at 12 h cells were fixed and processed for confocal microscopy. Phagosomes were isolated from post nuclear supernatants (PNS) of infected cells as described previously [29]. Samples were then fixed and probed as described below. The cya constructs were generated by PCR using specific primers (Table 1). Measurement of cAMP in cell lysates for adenylate cyclase fusion assays was performed using the Direct Cyclic AMP Enzyme Immunoassay kit (Assay Designs), as we described previously [10].

**Table 1:** Primers used in this study.

Primer Name	Primer Sequence (5’-3’)
AnkB A1 F*	ACCAATAGCTACCACTTAAA
AnkB A1 R*	TCGCTTTATATGCTGTTGTC
AnkB A2 F*	AAAGATAACTATGGTGATTC
AnkB A2 R*	GTGGTAGCTATTGGTCTGGG
AnkB A1A2 R*	GTGGTAGCTATTGGTTCGC
AnkB fbox F*	CAACAGCATATAAAGCGA
AnkB fbox R*	CTTTTTCATAGCAGCAAATAG
AnkB cya F	GGATCCTTATGAAAAAGAATTTTTTTTCTG
AnkB fbox cya F	GGATCCTTATGAAAAAGCAACAGCATATAAAG
AnkB cya R	CTGCAGTTAACAAACAAGGCACTTGCT
AnkB LP-AA sub F*	GCTGAGGAAACAATTGTCAATAC
AnkB LP-AA sub R*	AGCATCAGAAAAAAAATTCTTTTTC

*Primers modified with 5′-phosphorylation.

### Transfection of HEK-293 cells

The *ankB* gene was cloned into the mammalian expression vector, p3XFlag-CMV-10 (Sigma). To generate the *ankB-^9^L^10^P/AA* allele, the wild type p3XFlag-CMV-10 AnkB plasmid was used as a template for PCR based site directed mutagenesis. HEK-293 cells were grown to 80% confluency and then re-plated onto coverslips coated with poly-L lysine. After 16 h, HEK-293 monolayers were transfected with 0.5 µg plasmid DNA/well using Fugene HD reagent following the manufacturer's recommendations (Roche).

### Confocal laser scanning microscopy

Processing of infected cells for confocal microscopy was performed as we described previously [9]. Polyclonal rabbit anti-*L. pneumophila* anti-serum was detected by Alexa Fluor 488-conjugated donkey anti-rabbit IgG (Invitrogen, Carlsbad, CA). For attachment experiments, extracellular bacteria were differentially labeled by antibody, prior to permeabilization, followed by Alexa fluor-647 goat anti-rabbit antibody. Poly-ubiquitinated proteins were detected using anti-polyubiquitin FK1 antibody (BIOMOL International/Affiniti, Exeter, United Kingdom), followed by 555-conjugated goat anti-mouse IgM (Invitrogen, Carlsbad, CA). For ectopic expression experiments 3X-flag tagged proteins were labeled with either mouse monoclonal or rabbit polyclonal anti-flag antibodies (Sigma), followed by appropriate Alexa-fluor conjugated secondary antibodies (Invitrogen, Carlsbad, CA). For single cell analysis studies, bacteria were detected with polyclonal rabbit anti-*L. pneumophila* anti-serum and mouse anti-flag antibodies. The cells were examined with an Olympus FV1000 laser scanning confocal microscope as we described previously [9]. On average, 8–15 0.2 um serial Z sections of each image were captured and stored for further analyses, using Adobe photoshop 6.0.

### In vivo co-immunoprecipitation

HEK-293 cells were co-transfected with plasmids encoding 3X-Flag AnkB, AnkB^9^L^10^P/AA, BAP or SKP2 and SKP1-GST as described above using Fugene HD reagent. Following 24 h incubation, cells were lysed using M-PER (Thermo Scientific) following the manufacturer's instructions. Flag or GST tagged proteins were purified using anti-Flag M2 agarose (Sigma) or high affinity GST resin (Genscript) according to manufacturer's instructions. Purified supernatants were heated at 96°C for 5 minutes in sample buffer and subjected to 10.4–15% gradient SDS-PAGE gel electrophoresis and western blot using anti-Flag M2 (Sigma) and anti-GST (Abcam) antibodies. Immunoblots were visualized using SuperSignal West Femto substrate (Thermo Scientific).

### Silencing of host SKP1 in HEK 293 cells

HEK 293 cells were maintained in DMEM (Invitrogen, Carlsbad, CA) supplemented with 10% heat inactivated fetal bovine serum. For each transfection reaction, oligonucleotide RNAi-1 (GCAAGUCAAUUGUAUUAGCAGAAUA) and (UAUUCUGCUAAUACAAUUGACUUGC); RNAi-2 (UCACACUACUUGCAUGUAAAGAAUA) and (UAUUCUUUACAUGCAAGUAGUGUGA); or RNAi-3 (CAGAAAGCAUCCAUCAUGAAUGCAA) and (UUGCAUUCAUGAUGGAUGCUUUCUG) was diluted in oligofectamine and transfected for 4 hours as recommended (Invitrogen). Cells were washed and further incubated for 48 hours and infected as described above. To confirm silencing of SKP1 protein expression Western blot analyses were performed using standard procedures, as described above. Briefly, HEK-293 cells were lysed (Pierce) subjected to SDS-PAGE, and immunoblotted with the anti-SKP1 antibody.

### Infection of A/J mice with *L. pneumophila*

Female pathogen-free, 6–8 week old A/J mice were used for infection by intratracheal inoculation with 50 µl containing the bacterial dose as we described previously [36]. Mice were humanely euthanized at various times, the lungs were removed, homogenized and dilutions were cultured on BCYE agar for 72 h as described previously [36].

## Supporting Information

Figure S1 The F-box domain of AnkB shares similar alpha helix topology to mammalian F-box proteins. A: Amino acid alignment of the F-box domains of AnkB and the mammalian F-box proteins B-TrCP, CDC4 and SKP2. Alignments were performed using ClustalW software. Red residues represent conserved identical residues, while blue residues indicate conserved residues with similar biochemical properties. Boxed areas represent the known alpha helical regions in the mammalian F-box proteins [37]. Alpha helices for the AnkB F-box domain were predicted using PROF analysis [38]. B: Cartoon representation of the predicted F-box and two ANK domains of AnkB and the domain deletion mutant alleles used in this study.
(0.17 MB PDF)

Figure S2 AnkB is essential for acquisition of polyubiquitinated proteins by the LCV in *D. discoideum*. Cells were infected with the wild type (WT) *L. pneumophila* (Lpn) and the isogenic *dotA* or *ankB* mutants, or the *ankB* mutant harboring the WT *ankB* or mutant *ankB* alleles. Representative images of co-localization of the LCVs within infected *D. discoideum* (A) or in semi-purified LCVs (B) with polyubiquitinated proteins at 2h post-infection. The cells or LCVs were labeled with anti-Lpn antibody (green) and anti-polyubiquitin (red) and then analyzed by confocal microscopy. The arrow indicates heavy co-localization of polyubiquitin with the WT strain. Quantification of % co-localization of the LCVs with polyubiquitinated proteins at 2h is shown. The data represent analyses of 100 infected cells or LCVs and are representative of three independent experiments.
(0.16 MB PDF)

Figure S3 AnkB triggers recruitment of polyubiquitinated proteins upon bacterial attachment to *A. polyphaga*. Cells were infected with the wild type (WT) *L. pneumophila* (Lpn) and the isogenic *dotA* or *ankB* mutants, or the *ankB* mutant harboring the WT *ankB* or mutant *ankB* alleles. Representative confocal microscopy images of infected U937 cells for co-localization of attached extracellular bacteria with polyubiquitinated (PolyUb) proteins at 15 min post-infection is shown in panel A. The arrow indicates heavy co-localization of polyubiquitin with the WT strain. Prior to permeabilization, extracellular *L. pneumophila* were labeled with an anti-Lpn antibody (blue), resulting in a dual labeling of the extracellular bacteria (green and blue). Host polyubiquitin was labeled after permeabilization (red). B) Quantitation of the %co-localization of attached bacteria with polyubiquitinated proteins was determined at various time points by analysis of 100 attached bacteria. The data represent analyses of 100 infected cells and are representative of three independent experiments, and the error bars represent standard deviation.
(0.24 MB PDF)

Figure S4 The F-box domain of AnkB is essential for intracellular proliferation of *L. pneumophila* within hMDMs. Infection of hMDMs with WT and the isogenic mutants *dotA* and *ankB* and the *ankB* mutant harboring the *ankBΔF-box* allele was carried out in triplicate with an MOI of 10 for 1 h followed by 1 h gentamicin treatment to kill extracellular bacteria. The infected monolayers were lysed at different time points and plated onto agar plates for colony enumeration. The experiment was done 3 times and the data are representative of one independent experiment. Error bars represent standard deviation.
(0.11 MB PDF)

Figure S5 Rescue of the intracellular growth defect of the *ankB* mutant in HEK293 cells expressing AnkB. After 12 h post-infection of HEK-293 cells, 100 infected cells were analyzed by laser scanning confocal microscopy for formation of replicative phagosomes. Single cell analysis of replicative phagosomes in un-transfected (A) or 3X-Flag AnkB transfected (B) HEK-293 cells at 12h post-infection. Quantitative analyses in A and B were based on examination of 100 infected cells and the error bars represent standard deviation. The data are representative of three independent experiments.
(0.11 MB PDF)

## References

[1] Molmeret M, Horn M, Wagner M, Santic M, Abu Kwaik Y (2005). Amoebae as training grounds for intracellular bacterial pathogens.. Appl Environ Microbiol.

[2] Isberg RR, O'Connor TJ, Heidtman M (2009). The *Legionella pneumophila* replication vacuole: making a cosy niche inside host cells.. Nat Rev Microbiol.

[3] Shin S, Roy CR (2008). Host cell processes that influence the intracellular survival of *Legionella pneumophila*.. Cell Microbiol.

[4] Segal G, Purcell M, Shuman HA (1998). Host cell killing and bacterial conjugation require overlapping sets of genes within a 22-kb region of the *Legionella pneumophila* genome.. Proc Natl Acad Sci U S A.

[5] Vogel JP, Andrews HL, Wong SK, Isberg RR (1998). Conjugative transfer by the virulence system of *Legionella pneumophila*.. Science.

[6] de Felipe KS, Glover RT, Charpentier X, Anderson OR, Reyes M (2008). *Legionella* eukaryotic-like type IV substrates interfere with organelle trafficking.. PLoS Pathog.

[7] Habyarimana F, Price CT, Santic M, Al-Khodor S, Kwaik YA Molecular characterization of the Dot/Icm-translocated AnkH and AnkJ eukaryotic-like effectors of *Legionella pneumophila*.. Infect Immun. In press..

[8] Al-Khodor S, Price CTD, Kalia A, Abu Kwaik Y Microbial Eukaryotic-like Ankyrins: conserved structures with functional diversity to modulate host-pathogen interactions.. Trends Microbiol. In press.

[9] Habyarimana F, Al-Khodor S, Kalia A, Graham JE, Price CT (2008). Role for the Ankyrin eukaryotic-like genes of *Legionella pneumophila* in parasitism of protozoan hosts and human macrophages.. Environ Microbiol.

[10] Al-Khodor S, Price CT, Habyarimana F, Kalia A, Abu Kwaik Y (2008). A Dot/Icm-translocated ankyrin protein of *Legionella pneumophila* is required for intracellular proliferation within human macrophages and protozoa.. Mol Microbiol.

[11] Liu Y, Luo ZQ (2007). The *Legionella pneumophila* effector SidJ is required for efficient recruitment of endoplasmic reticulum proteins to the bacterial phagosome.. Infect Immun.

[12] Petroski MD, Deshaies RJ (2005). Function and regulation of cullin-RING ubiquitin ligases.. Nat Rev Mol Cell Biol.

[13] Kerscher O, Felberbaum R, Hochstrasser M (2006). Modification of proteins by ubiquitin and ubiquitin-like proteins.. Annu Rev Cell Dev Biol.

[14] Schulman BA, Carrano AC, Jeffrey PD, Bowen Z, Kinnucan ER (2000). Insights into SCF ubiquitin ligases from the structure of the Skp1-Skp2 complex.. Nature.

[15] Russell ID, Grancell AS, Sorger PK (1999). The unstable F-box protein p58-Ctf13 forms the structural core of the CBF3 kinetochore complex.. J Cell Biol.

[16] Kondo-Okamoto N, Ohkuni K, Kitagawa K, McCaffery JM, Shaw JM (2006). The novel F-box protein Mfb1p regulates mitochondrial connectivity and exhibits asymmetric localization in yeast.. Mol Biol Cell.

[17] Neves AM, Guerreiro P, Rodrigues-Pousada C (1991). The macronuclear polyubiquitin gene of the ciliate *Tetrahymena pyriformis*.. DNA Seq.

[18] Hu Q, Henney HR (1997). An *Acanthamoeba* polyubiquitin gene and application of its promoter to the establishment of a transient transfection system.. Biochim Biophys Acta.

[19] Ennis HL, Dao DN, Pukatzki SU, Kessin RH (2000). *Dictyostelium amoebae* lacking an F-box protein form spores rather than stalk in chimeras with wild type.. Proc Natl Acad Sci U S A.

[20] Ennis HL, Dao DN, Wu MY, Kessin RH (2003). Mutation of the *Dictyostelium fbxA* gene affects cell-fate decisions and spatial patterning.. Protist.

[21] Mohanty S, Lee S, Yadava N, Dealy MJ, Johnson RS (2001). Regulated protein degradation controls PKA function and cell-type differentiation in *Dictyostelium*.. Genes Dev.

[22] Chung CY, Reddy TB, Zhou K, Firtel RA (1998). A novel, putative MEK kinase controls developmental timing and spatial patterning in *Dictyostelium* and is regulated by ubiquitin-mediated protein degradation.. Genes Dev.

[23] West CM, Kozarov E, Teng-umnuay P (1997). The cytosolic glycoprotein FP21 of Dictyostelium discoideum is encoded by two genes resulting in a polymorphism at a single amino acid position.. Gene.

[24] Teng-umnuay P, Morris HR, Dell A, Panico M, Paxton T (1998). The cytoplasmic F-box binding protein SKP1 contains a novel pentasaccharide linked to hydroxyproline in Dictyostelium.. J Biol Chem.

[25] Kobe B, Kajava AV (2001). The leucine-rich repeat as a protein recognition motif.. Curr Opin Struct Biol.

[26] Smith TF, Gaitatzes C, Saxena K, Neer EJ (1999). The WD repeat: a common architecture for diverse functions.. Trends Biochem Sci.

[27] Veiga E, Cossart P (2005). Ubiquitination of intracellular bacteria: a new bacteria-sensing system?. Trends Cell Biol.

[28] Dorer MS, Kirton D, Bader JS, Isberg RR (2006). RNA interference analysis of *Legionella* in *Drosophila* cells: exploitation of early secretory apparatus dynamics.. PLoS Pathog.

[29] Conover GM, Derre I, Vogel JP, Isberg RR (2003). The *Legionella pneumophila* LidA protein: a translocated substrate of the Dot/Icm system associated with maintenance of bacterial integrity.. Mol Microbiol.

[30] Kubori T, Hyakutake A, Nagai H (2008). *Legionella* translocates an E3 ubiquitin ligase that has multiple U-boxes with distinct functions.. Mol Microbiol.

[31] Willems AR, Schwab M, Tyers M (2004). A hitchhiker's guide to the cullin ubiquitin ligases: SCF and its kin.. Biochim Biophys Acta.

[32] Ivanov SS, Roy CR (2009). Modulation of ubiquitin dynamics and suppression of DALIS formation by the *Legionella pneumophila* Dot/Icm system.. Cell Microbiol.

[33] Amor JC, Swails J, Zhu X, Roy CR, Nagai H (2005). The structure of RalF, an ADP-ribosylation factor guanine nucleotide exchange factor from *Legionella pneumophila*, reveals the presence of a cap over the active site.. J Biol Chem.

[34] Cazalet C, Rusniok C, Bruggemann H, Zidane N, Magnier A (2004). Evidence in the *Legionella pneumophila* genome for exploitation of host cell functions and high genome plasticity.. Nat Genet.

[35] de Felipe KS, Pampou S, Jovanovic OS, Pericone CD, Ye SF (2005). Evidence for acquisition of *Legionella* type IV secretion substrates via interdomain horizontal gene transfer.. J Bacteriol.

[36] Santic M, Asare R, Doric M, Abu Kwaik Y (2007). Host-dependent trigger of caspases and apoptosis by *Legionella pneumophila*.. Infect Immun.

[37] Sonnberg S, Seet BT, Pawson T, Fleming SB, Mercer AA (2008). Poxvirus ankyrin repeat proteins are a unique class of F-box proteins that associate with cellular SCF1 ubiquitin ligase complexes.. Proc Natl Acad Sci U S A.

[38] Rost B, Yachdav G, Liu J (2004). The PredictProtein server.. Nucleic Acids Res.

