# Supplementary material for: Molecular Mimicry by an F-Box Effector of Legionella pneumophila Hijacks a Conserved Polyubiquitination Machinery within Macrophages and Protozoa
Source: PLoS Pathog. 2009 Dec 24;5(12):e1000704. doi: 10.1371/journal.ppat.1000704 (PMC2790608; doi:10.1371/journal.ppat.1000704)
Supplement: Figure S1 — The F-box domain of AnkB shares similar alpha helix topology to mammalian F-box proteins. A: Amino acid alignment of the F-box domains of AnkB and the mammalian F-box proteins B-TrCP, CDC4 and SKP2. Alignments were performed using ClustalW software. Red residues represent conserved identical residues, while blue residues indicate conserved residues with similar biochemical properties. Boxed areas represent the known alpha helical regions in the mammalian F-box proteins [37]. Alpha helices for the AnkB F-box domain were predicted using PROF analysis [38]. B: Cartoon representation of the predicted F-box and two ANK domains of AnkB and the domain deletion mutant alleles used in this study. (0.17 MB PDF) [file ppat.1000704.s001.pdf]

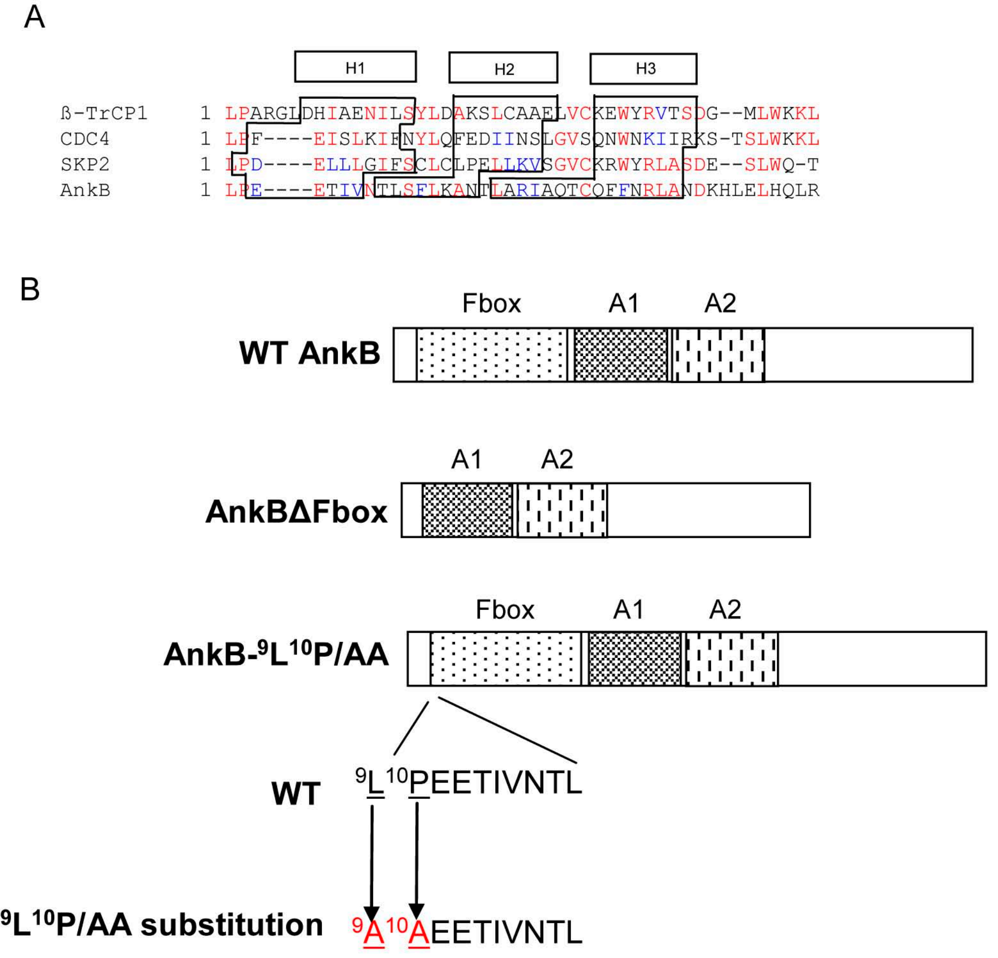
A
H1
H2
H3
ß-TrCP1 1 LPARGLDHIAENILSYLDAKSLCAAELVCKEWYRVTSDG--MLWKKL
CDC4 1 LPF----EISLKIFNYLQFEDIINSLGVSQNWNKIIRKS-TSLWKKL
SKP2 1 LPD----ELLLGIFSCLCLPELLKVSGVCKRWYRLASDE--SLWQ-T
AnkB 1 LPE----ETIVNTLSFLKANTLARIAQTCQFFNRLANDKHLELHQLR
B
Fbox
A1
A2
WT AnkB
A1
A2
AnkBΔFbox
Fbox
A1
A2
AnkB-9L10P/AA
WT
9L 10PEETIVNTL
9L10P/AA substitution
9A 10AEETIVNTL

Fig. S1
