# Supplementary figures and images for: Molecular Mimicry by an F-Box Effector of Legionella pneumophila Hijacks a Conserved Polyubiquitination Machinery within Macrophages and Protozoa
Source: PLoS Pathog. 2009 Dec 24;5(12):e1000704. doi: 10.1371/journal.ppat.1000704 (PMC2790608; doi:10.1371/journal.ppat.1000704)

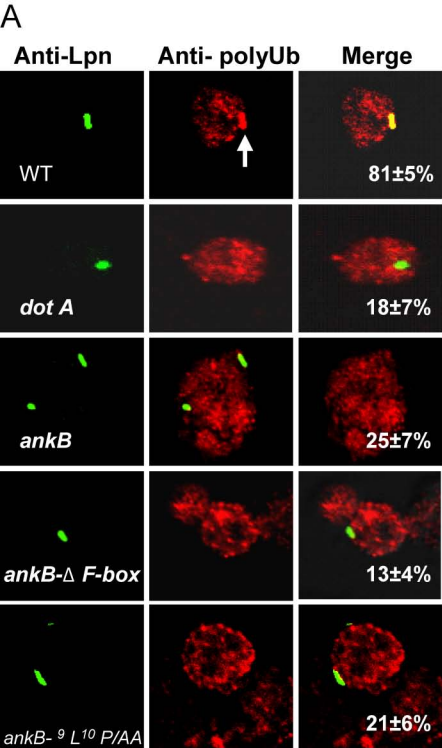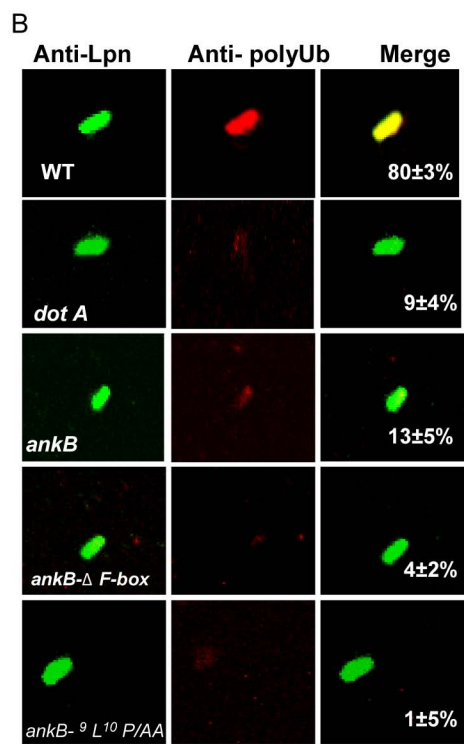

Fig. S2

Supplement: Figure S2 — AnkB is essential for acquisition of polyubiquitinated proteins by the LCV in D. discoideum. Cells were infected with the wild type (WT) L. pneumophila (Lpn) and the isogenic dotA or ankB mutants, or the ankB mutant harboring the WT ankB or mutant ankB alleles. Representative images of co-localization of the LCVs within infected D. discoideum (A) or in semi-purified LCVs (B) with polyubiquitinated proteins at 2h post-infection. The cells or LCVs were labeled with anti-Lpn antibody (green) and anti-polyubiquitin (red) and then analyzed by confocal microscopy. The arrow indicates heavy co-localization of polyubiquitin with the WT strain. Quantification of % co-localization of the LCVs with polyubiquitinated proteins at 2h is shown. The data represent analyses of 100 infected cells or LCVs and are representative of three independent experiments. (0.16 MB PDF) [file ppat.1000704.s002.pdf]

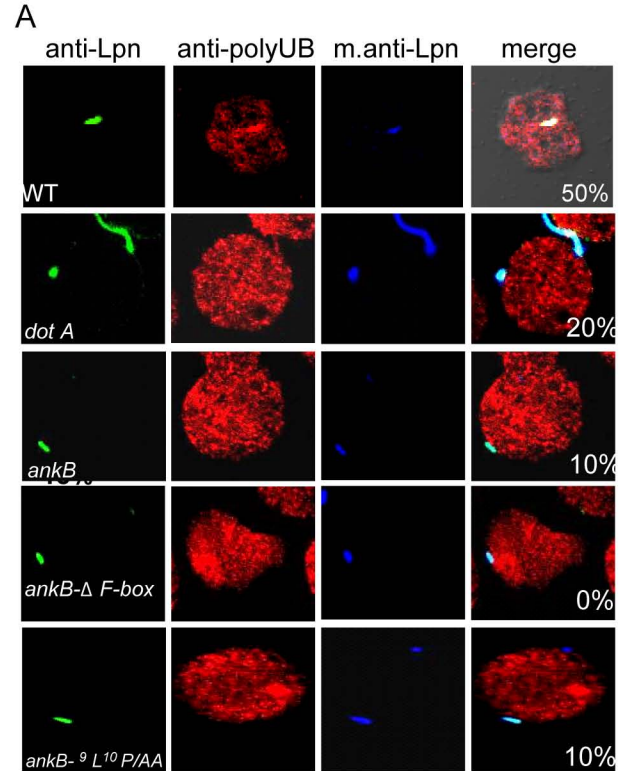

**B**

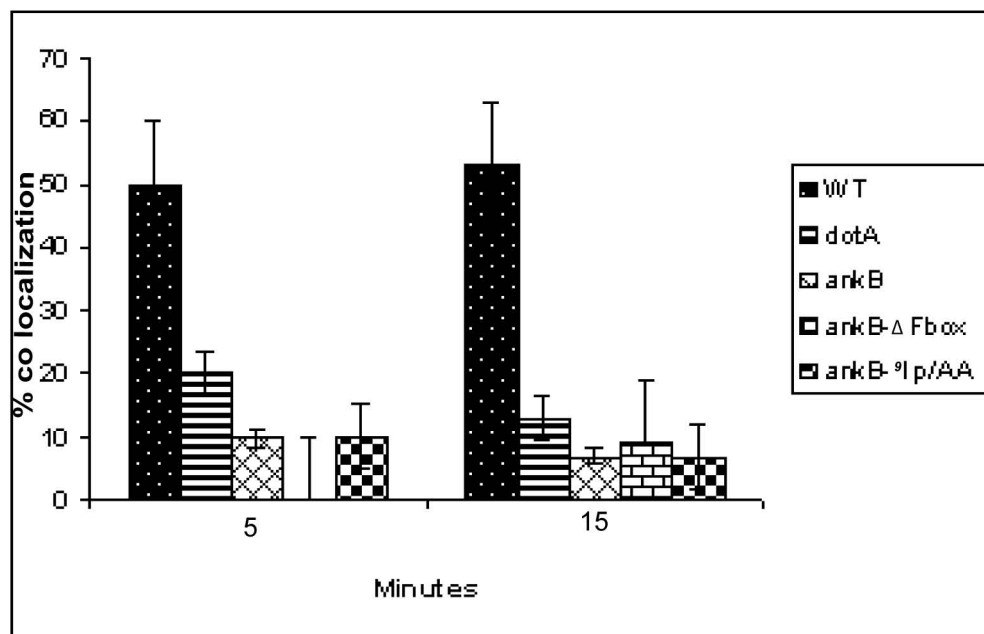

Fig. S3

Supplement: Figure S3 — AnkB triggers recruitment of polyubiquitinated proteins upon bacterial attachment to A. polyphaga. Cells were infected with the wild type (WT) L. pneumophila (Lpn) and the isogenic dotA or ankB mutants, or the ankB mutant harboring the WT ankB or mutant ankB alleles. Representative confocal microscopy images of infected U937 cells for co-localization of attached extracellular bacteria with polyubiquitinated (PolyUb) proteins at 15 min post-infection is shown in panel A. The arrow indicates heavy co-localization of polyubiquitin with the WT strain. Prior to permeabilization, extracellular L. pneumophila were labeled with an anti-Lpn antibody (blue), resulting in a dual labeling of the extracellular bacteria (green and blue). Host polyubiquitin was labeled after permeabilization (red). B) Quantitation of the %co-localization of attached bacteria with polyubiquitinated proteins was determined at various time points by analysis of 100 attached bacteria. The data represent analyses of 100 infected cells and are representative of three independent experiments, and the error bars represent standard deviation. (0.24 MB PDF) [file ppat.1000704.s003.pdf]

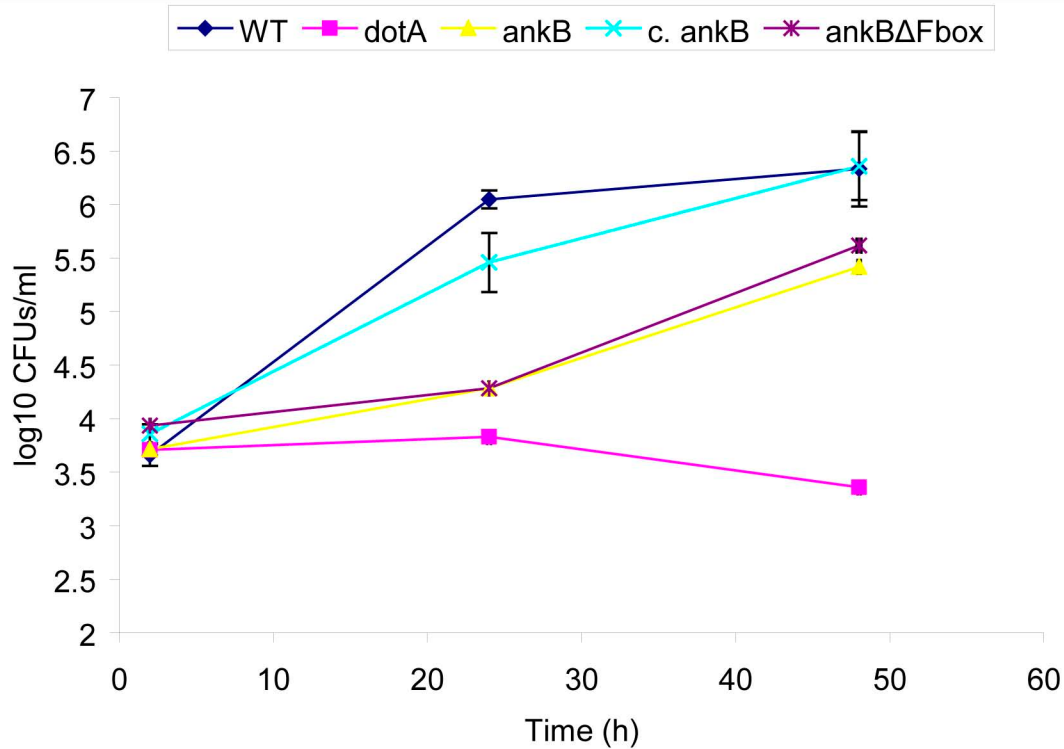

Fig. S4

Supplement: Figure S4 — The F-box domain of AnkB is essential for intracellular proliferation of L. pneumophila within hMDMs. Infection of hMDMs with WT and the isogenic mutants dotA and ankB and the ankB mutant harboring the ankBΔF-box allele was carried out in triplicate with an MOI of 10 for 1 h followed by 1 h gentamicin treatment to kill extracellular bacteria. The infected monolayers were lysed at different time points and plated onto agar plates for colony enumeration. The experiment was done 3 times and the data are representative of one independent experiment. Error bars represent standard deviation. (0.11 MB PDF) [file ppat.1000704.s004.pdf]

A

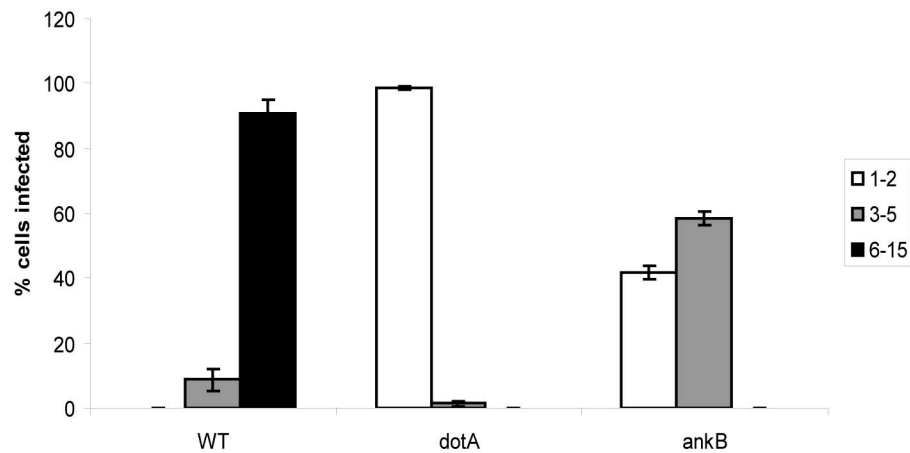

B

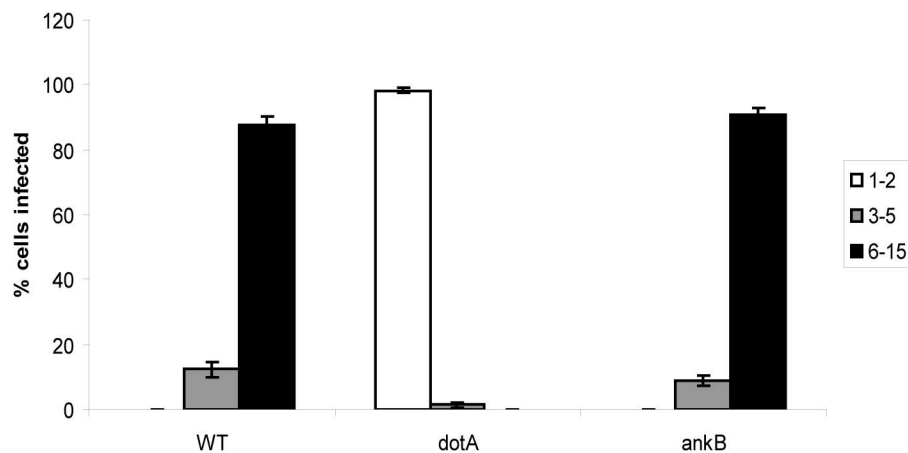

Fig. S5

Supplement: Figure S5 — Rescue of the intracellular growth defect of the ankB mutant in HEK293 cells expressing AnkB. After 12 h post-infection of HEK-293 cells, 100 infected cells were analyzed by laser scanning confocal microscopy for formation of replicative phagosomes. Single cell analysis of replicative phagosomes in un-transfected (A) or 3X-Flag AnkB transfected (B) HEK-293 cells at 12h post-infection. Quantitative analyses in A and B were based on examination of 100 infected cells and the error bars represent standard deviation. The data are representative of three independent experiments. (0.11 MB PDF) [file ppat.1000704.s005.pdf]
